# Effect of temperature on accidental human mortality: A time-series analysis in Shenzhen, Guangdong Province in China

**DOI:** 10.1038/s41598-020-65344-y

**Published:** 2020-05-21

**Authors:** Tingyu Lian, Yingbin Fu, Mingwei Sun, Mingjuan Yin, Yan Zhang, Lingfeng Huang, Jingxiao Huang, Ziqian Xu, Chen Mao, Jindong Ni, Gang Liu

**Affiliations:** 1 Department of Epidemiology and Biostatistics, Dongguan Key Laboratory of Environmental Medicine, School of Public Health, Key Laboratory of Precision Public Health, Guangdong Medical University, 1 Xincheng Avenue Songshanhu District, Dongguan, 523000 Guangdong China; 2 Shenzhen Center for Disease Control and Prevention, 8 Longyuan Road, Longzhu Avenue Nanshan District, Shenzhen, 518000 Guangdong China; 3 Department of Epidemiology, School of Public Health, Southern Medical University, Guangzhou, 510515 Guangdong China

**Keywords:** Environmental impact, Risk factors

## Abstract

Health-risk assessments of temperature are central to determine total non-accidental human mortality; however, few studies have investigated the effect of temperature on accidental human mortality. We performed a time-series study combined with a distributed lag non-linear model (DLNM) to quantify the non-linear and delayed effects of daily mean temperature on accidental human mortality between 2013 and 2017 in Shenzhen, China. The threshold for effects of temperature on accidental human mortality occurred between 5.6 °C and 18.5 °C. Cold exposures, but not hot exposures, were significantly associated with accidental human mortality. All of the observed groups were susceptible to cold effects, with the strongest effects presented in females (relative risk [RR]: 3.14, 95% confidence interval (CI) [1.44–6.84]), followed by poorly educated people (RR: 2.63, 95% CI [1.59–4.36]), males (RR: 1.79, 95% CI [1.10–2.92]), and well-educated people (RR: 1.20, 95% CI [0.58–2.51]). Pooled estimates for cold effects at a lag of 0–21 days (d) were also stronger than hot effects at a lag of 0–2 d. Our results indicate that low temperatures increased the risk of accidental human mortality. Females and poorly educated people were more susceptible to the low temperatures. These findings imply that interventions which target vulnerable populations during cold days should be developed to reduce accidental human mortality risk.

## Introduction

Along with the climate change, the effect of temperature on human mortality remains an issue of increasing public health significance worldwide^1^. The considerable temperature impact on human mortality have been extensively reported in many countries at global. For example, a global study including 306 communities across twelve countries /regions with varies climate patterns revealed both cold and hot temperatures increased the risk of non-accidental human mortality^2^. A national study of mortality including 12 large cities in China found that 1 °C increase of moderately high temperature is associated with 4.6%, 4.2%, and 6.3% in mortality of non-accidental cause, circulatory disease and respiratory disease, respectively^3^. The proportion of temperature-related stroke death was 17.7% in southern China^4^. These findings highlight the adverse effects of temperature on non-accidental human mortality. However, most of previous studies on this subject have been focus on examining the effects of temperature on non-accidental human mortality and cause-specific human mortality, few studies have quantified the effects of temperature on accidental human mortality, especially in China, despite growing awareness of human vulnerability to weather change.

Accidental human mortality was characterized by unexpected causes, and unintentional events or behavior, including transport accidents and other external causes of accidental injury, such as falls, accidental drowning and submersion, accidental poisoning. It is a well-recognized major cause of death and contributes significantly to health care costs and the burden on society. In China, accidental human mortality is the fourth leading cause of death (54.0 deaths per 100,000 person-years)^5^. Accidental human mortality accounts for 3.48% of infant deaths in Guangzhou of Guangdong Province in China^6^. One study analyzed injury-related mortality on life expectancy in China, and showed that the greatest impact on life expectancy was accidents from traffic injuries (0.29 years lost overall), followed by falls or drownings^7^. The government has been paying greater attention to the prevention of accidents in Shenzhen. For example, the strictest traffic regulations in China have been implemented in Shenzhen and people who do not stop at red lights will be fined. The “Anti-fall Project for the Elderly” in Luohu, Shenzhen involved the installation of anti-fall handrails and the placing of anti-slip mats for the elderly to reduce the occurrence of falls.

The work in the present study was motivated by our observation that there is limited information regarding relationship between temperature and accidental human mortality. Since many of the impacts of temperature exposure can be largely prevented if government and individuals take effective precautionary measures. Therefore, we argue that there is an ongoing need to quantify the effect of temperature on accidental human mortality, as well as complement the development of temperature-related health studies. In our study, we used the DLNM to assess the effects of daily mean temperature on accidental human mortality in Shenzhen, China, and also determined whether or not sociodemographic status (e.g. gender and level of education) has an effect.

## Materials and methods

### Data collection

**Figure 1:**
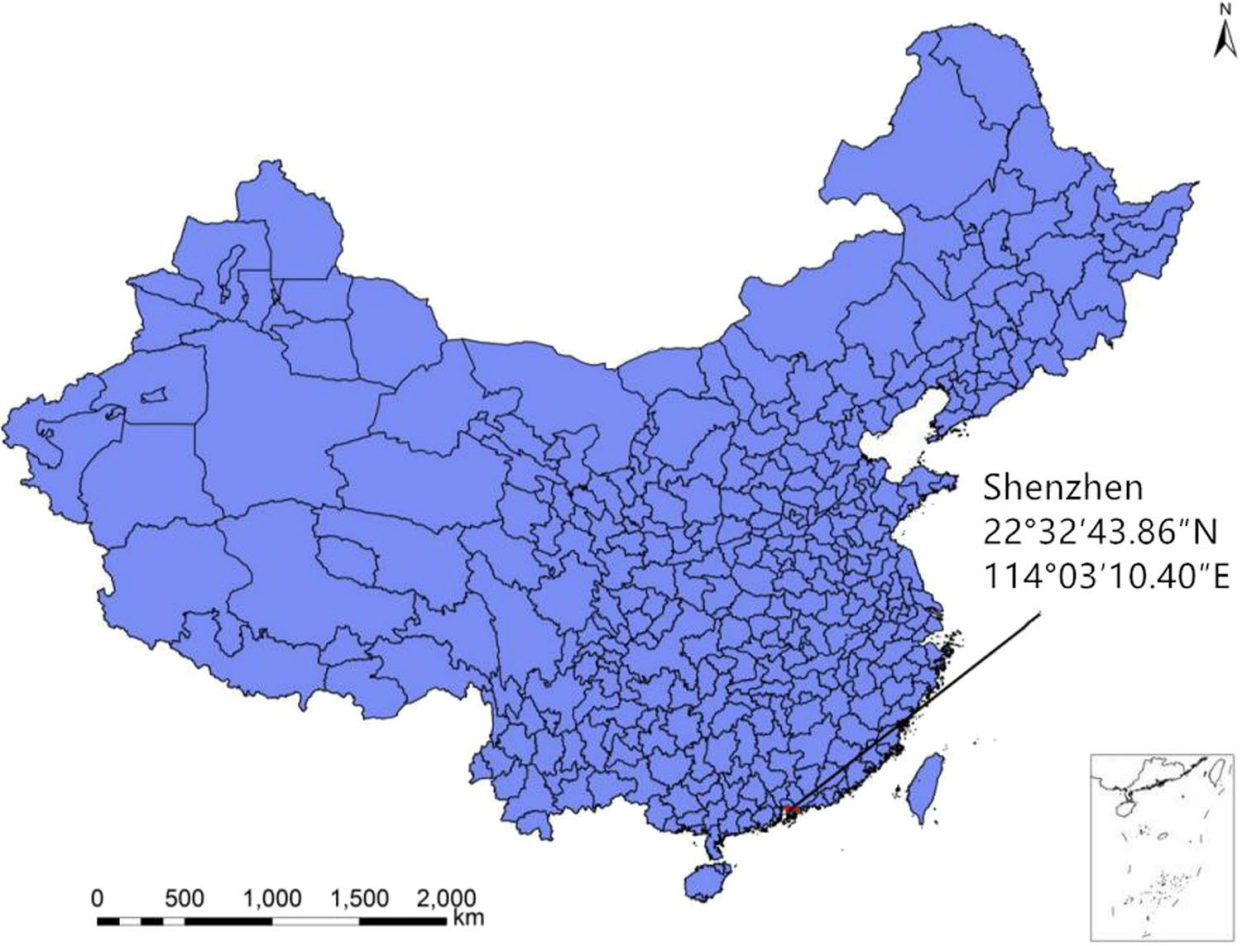
Geographical distribution of Shenzhen.

Shenzhen is one of China’s economic centers, known for its “Reform and Openness” policies with the outside world. It is a major city located in south Guangdong Province, linking Hong Kong to the mainland, and has a subtropical climate that is characterized by long summers and short winters with abundant sunshine (Fig. 1).

All accidental death data were obtained based on the national Death Surveillance Point System (DSPS) between 2013 and 2017. For each death case, physicians complete the medical certificate of death and provided demographic information in accordance with the requirements of the National Code for the Registration and Reporting of Causes of Death, and report the death cases online through DSPS. The centers for Disease Control and Prevention is responsible for auditing and identifying the causes of death and coding the causes of death in accordance with the rules of the International Classification of Diseases 10th Revision (ICD-10). The ICD is a global standard diagnostic classification for mortality and morbidity statistics maintained by the World Health Organization (WHO). Currently ICD-10 is the major revision, which has inherited a structure from previous versions of ICD. We classified accidental human mortality (ICD-10 V01-X59) using the ICD-10, including transport accidents (V01-V99) and other external causes of accidental injury(W00-X59). The data were validated by the Shenzhen Centre for Disease Control and Prevention.

Daily weather data were obtained from the Shenzhen Municipal Meteorological Bureau, including daily mean temperature (°C), relative humidity (%), wind speed (m/s), precipitation (mm), pressure (hPa), and sunshine (h). In our study, we adopted the daily mean temperature to evaluate the effects of temperature on accidental human mortality, as the daily mean temperature represents the temperature exposure for both day and night^8^.

### Data analysis

Descriptive statistics were calculated for meteorological variables and accidental death data. Non-linear and delayed effects of temperature on accidental human mortality were identified using the DLNM, which specified by the cross-basis function that describes a bi-dimensional functional space expressed by the combination of basic functions of temperature and lags. DLNM has become a powerful tool to investigate the health effects of temperature^9–11^. We applied a quasi-Poisson regression model combined with DLNM to determine the influence of daily mean temperature on accidental human mortality^12^. All possible confounding meteorological variables, including humidity, wind speed, sunshine, pressure, and precipitation, were included in the model. The model is given as follows:

**Yt ~Poisson (µt)**

1 $$\begin{array}{c}{\bf{L}}{\bf{o}}{\bf{g}}({{\bf{u}}}_{{\bf{t}}})={\boldsymbol{\alpha }}+{\boldsymbol{\beta }}{\bf{T}}{\bf{e}}{\bf{m}}{{\bf{p}}}_{{\bf{t}},{\bf{l}}}+{\bf{n}}{\bf{s}}({\bf{t}}{\bf{i}}{\bf{m}}{\bf{e}},{\bf{7}}\times {\bf{5}})+{\bf{n}}{\bf{s}}({\bf{p}}{\bf{r}}{\bf{e}}{\bf{c}}{\bf{i}}{\bf{p}}{\bf{i}}{\bf{t}}{\bf{a}}{\bf{t}}{\bf{i}}{\bf{o}}{{\bf{n}}}_{{\bf{t}}},{\bf{3}})+\\ \,{\bf{n}}{\bf{s}}({\bf{p}}{\bf{r}}{\bf{e}}{\bf{s}}{\bf{s}}{\bf{u}}{\bf{r}}{{\bf{e}}}_{{\bf{t}}},{\bf{3}})+{\bf{n}}{\bf{s}}({\bf{h}}{\bf{u}}{\bf{m}}{\bf{i}}{\bf{d}}{\bf{i}}{\bf{t}}{{\bf{y}}}_{{\bf{t}}},{\bf{3}})+{\bf{n}}{\bf{s}}({\bf{w}}{\bf{i}}{\bf{n}}{\bf{d}}{\bf{s}}{\bf{p}}{\bf{e}}{\bf{e}}{{\bf{d}}}_{{\bf{t}}},{\bf{3}})+{\bf{n}}{\bf{s}}({\bf{s}}{\bf{u}}{\bf{n}}{\bf{s}}{\bf{h}}{\bf{i}}{\bf{n}}{{\bf{e}}}_{{\bf{t}}},{\bf{3}})+{\boldsymbol{\eta }}{\bf{D}}{\bf{o}}{\bf{w}}{\bf{t}}\end{array}$$

where t is the day of the observation, µ_t_ is the expected death count on day t, Y_t_ is the observed daily number of deaths at calendar day t (t = 1,2,3…1826), α is the intercept, and Temp_t,l_ was the cross-basis matrix for temperature produced by the DLNM. β is a vector of coefficients for Temp_t,l_, and l is the lag days, ns indicates the natural cubic spline (ns), Dowt represents the day of the week (Dow) on day t, and η is a vector of coefficients. Based on the vector of estimated coefficient β in Equation 1, the DLNM was used to predict the effects and standard errors for combinations of temperature and lags.

Seven degrees of freedom (df) per year for calendar day by using a natural cubic spline were used to take account of seasonality and long-term trends in accordance with previous studies^13^. To choose the df for temperature and lag, we chose 5 df for temperature and 3 df for lag by the minimum of the Akaike information criterion (AIC) value^9,11,14^. A natural cubic spline of three df for humidity, wind speed, sunshine, pressure, and precipitation were used by the combination of df that could minimize the AIC value. In the cross-basis function, three internal knots were placed at the 10th, 50th, and 90th percentiles of the temperature distribution, with a maximum lag of up to 21 d^2,14,15^. The median value of temperature (24.7 °C) was defined as the baseline temperature (centering value) for calculating the RR^1,8,12^. For relative temperature changes, extreme cold and hot were defined as temperatures below the 5th percentile (14 °C) and above the 95th percentile (30 °C) of the daily mean temperature. We plotted the relative risk against temperature and lags and also plotted overall effect of temperature on accidental human mortality summed over lag days. In addition, we refer to levels of education as a socioeconomic indicator: ≤9 years of schooling was defined as a poor education; and >9 years was defined as a good education^16^.

A sensitivity analysis was conducted by exploring changing the df from 7 to 9 for time trends and the maximum lag days (14, 21, and 30) for the DLNM to test the robustness of our results. R (version 3.5.1) was used to fit all the models in this study. The distributed lag non-linear model (dlnm) package for the DLNM was used in the analysis.

### Ethics approval and consent to participate

Date were collected for administrative purposes, included no identifiable private information in this research. According to the Ethical Review of Biomedical Research Involving Human Beings, determined as not acquired from human subjects. Since the study used only the surveillance data collected by the Shenzhen Center for Disease Prevention and Control, inform consent to participate was not required. The Ethical consideration has been waived by the Shenzhen Centre for Disease Control and Prevention Ethical Review Committee due to the nature of the study.

## Results

A summary of average daily accidental human mortality and meteorological indicator levels in Shenzhen from 2013 to 2017 are presented in Table 1. Mean values, and 25th, 50th, 75th, and 95th percentiles for daily death counts, as well as mean values of meteorological data, are provided. The average daily mean temperature in Shenzhen was 23.45 °C during the study period. There were 4141 accidental deaths between January 2013 and December 2017, with 74.2% occurring in males. The average daily number of accidental deaths was 2.26. Death counts for different sub-groups varied significantly. The completeness of the data was good, with only 2.9% missing data in relation to level of education.

**Table 1:** Summary statistics of daily weather conditions and accidental human mortality in Shenzhen, China, 2013–2017.

**Variable**	**Number of observation**	**Mean** ± **SD**	**P25**	**P50**	**P75**	**P95**
**Accidental mortality**	4141	2.26 ± 2.41	1	2	3	6
Male	3072	1.68 ± 1.81	1	1	2	4
Female	1069	0.59 ± 0.99	0	0	1	2
**Education attainmen**t						
Poor education	3130	1.71 ± 2.14	1	1	2	4
Good education	887	0.49 ± 0.75	0	0	1	2
**Meteorological data**						
Temperature (°C)	1826	23.45 ± 5.49	19.10	24.70	28.10	30.30
Mean humidity (%)	1826	77.31 ± 13.34	71	79	87	96
Wind speed (m/s)	1826	1.52 ± 0.64	1.10	1.40	1.80	2.80
Precipitation (mm)	1826	5.39 ± 16.69	0	0	1.40	32.10
Pressure (hPa)	1826	999.33 ± 81.55	1001.0	1005.5	1010.90	1016.26
Sunshine (h)	1826	5.71 ± 0.64	2.50	6.10	8.73	10.80

**Figure 2:**
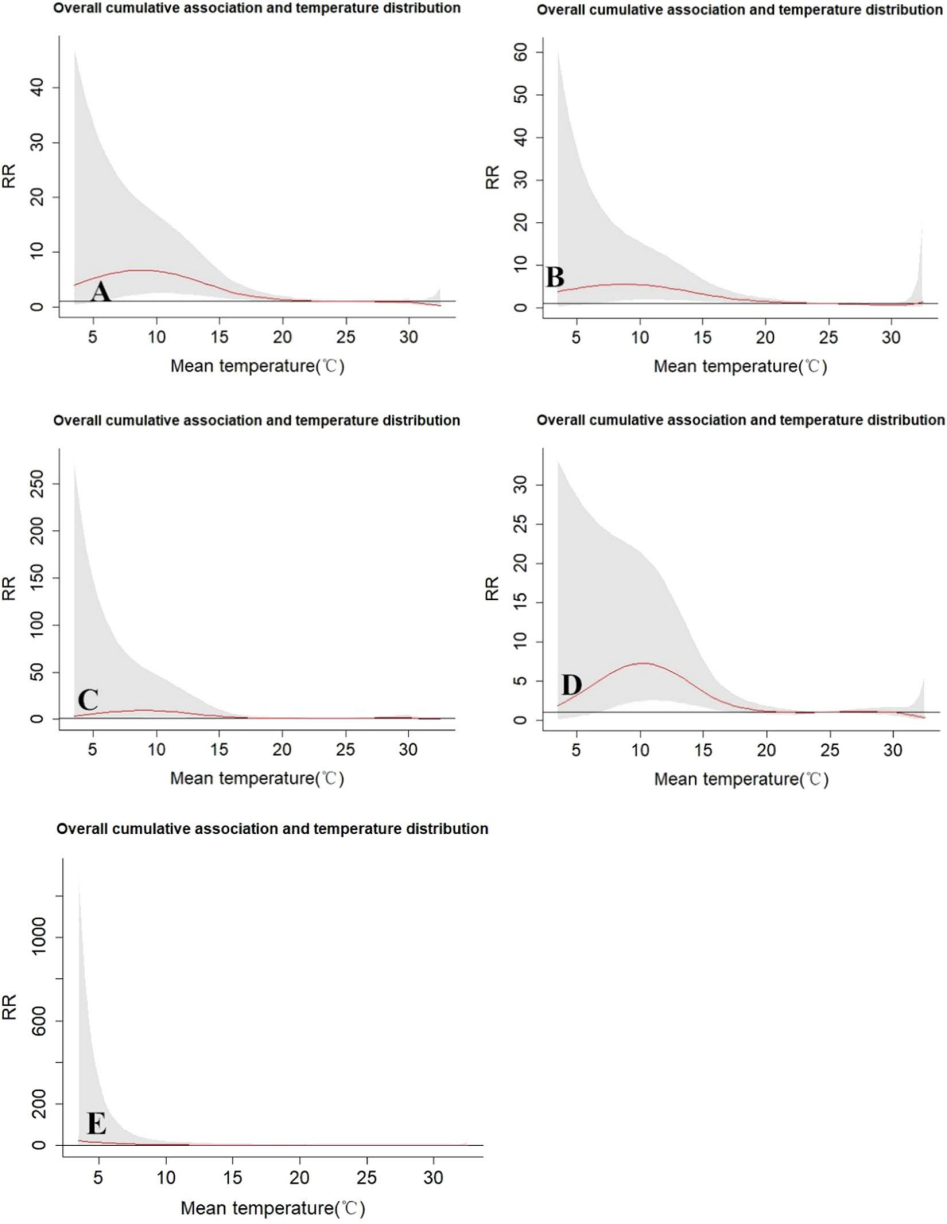
The estimated overall effects of mean temperature (°C) over 21 days on mortality types. The red lines are the mean relative risks, and the grey regions are 95%CI. (**A**) Accidental, (**B**) Male, (**C**) Female, (**D**) Poor education, **(E**) Good education. RR represents as the relative risk.

SD: standard deviation; Mean: daily average number of variables; P25, P50, P75, P95 are equal to the 25th, 50th, 75th, 95th percentile of the distributions;

In general, the effect of temperature on accidental human mortality demonstrated a non-linear and inverted U-shaped curve trend, with RR remaining significant during low temperatures, but not at hot temperatures (Fig. 2). The effects of temperatures became apparent at 5.6–18.5 °C, with peak levels occurring at 8.9 °C (RR: 6.72, 95% CI [2.37–19.03]). Analysis of subgroups demonstrated that the maximum mortality temperature observed was 8.8 °C for males (RR: 5.50, 95% CI [1.69–17.80]), 9.1 °C for females (RR: 9.51, 95% CI [1.64–55.9]), 10.2 °C for poorly educated people (RR: 10.2, 95% CI [2.47–21.3]), and 7.0 °C for well-educated people (RR: 8.92, 95% CI [1.007–79.09]). Risk decreased linearly for low temperatures below and above the maximum mortality temperature. To summarize, there was a risk of accidental human mortality at low temperatures, but not at hot temperatures.

**Figure 3:**
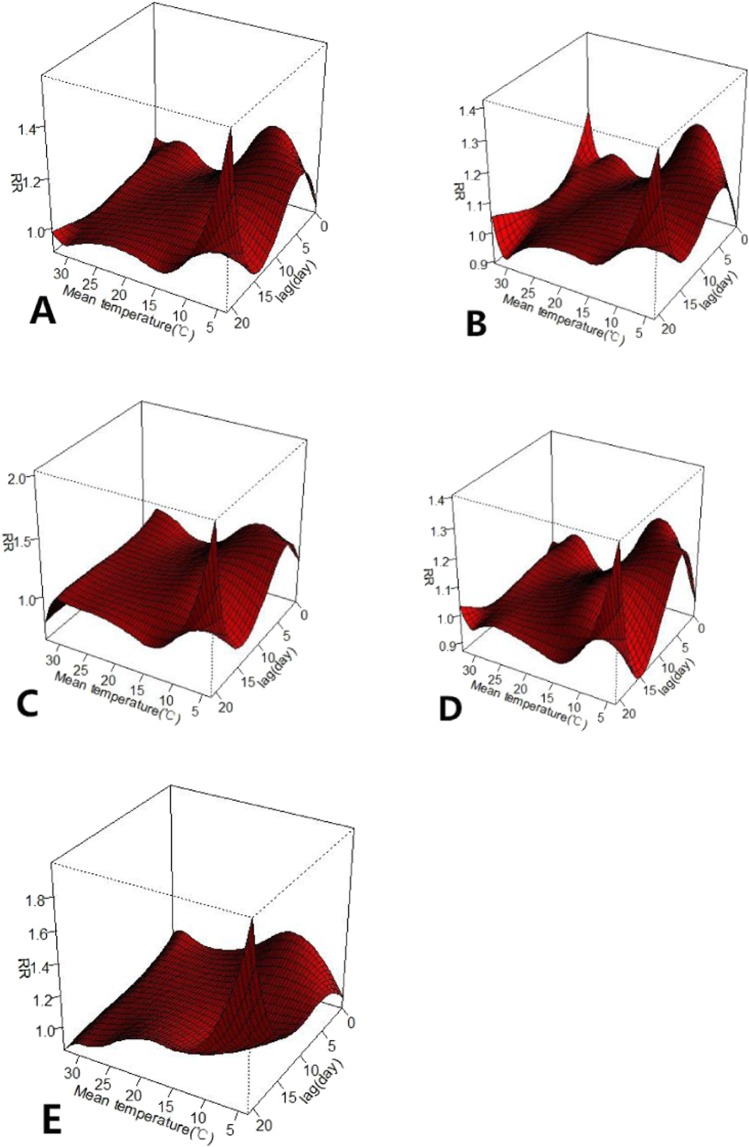
The relative risk of mortality types by mean temperature (°C). (**A**) Accidental, (**B**) Male, (**C**) Female, (**D**) Poor education, (**E**) Good education. RR represents as the relative risk.

The three-dimensional plots show the entire surface between the mean temperature and mortality categories at all lag days are shown in Fig. 3. The estimated effects of temperature were non-linear for all accidental human mortality types, with a higher RR at low temperatures shown to be significantly negatively correlated with accidental human mortality. Obvious lagged effects were present for low temperatures. In contrast, hot temperatures had no significant effects on accidental human mortality. The results indicate that when the temperature was colder, the lag time was longer, and a strong effect appeared.

**Figure 4:**
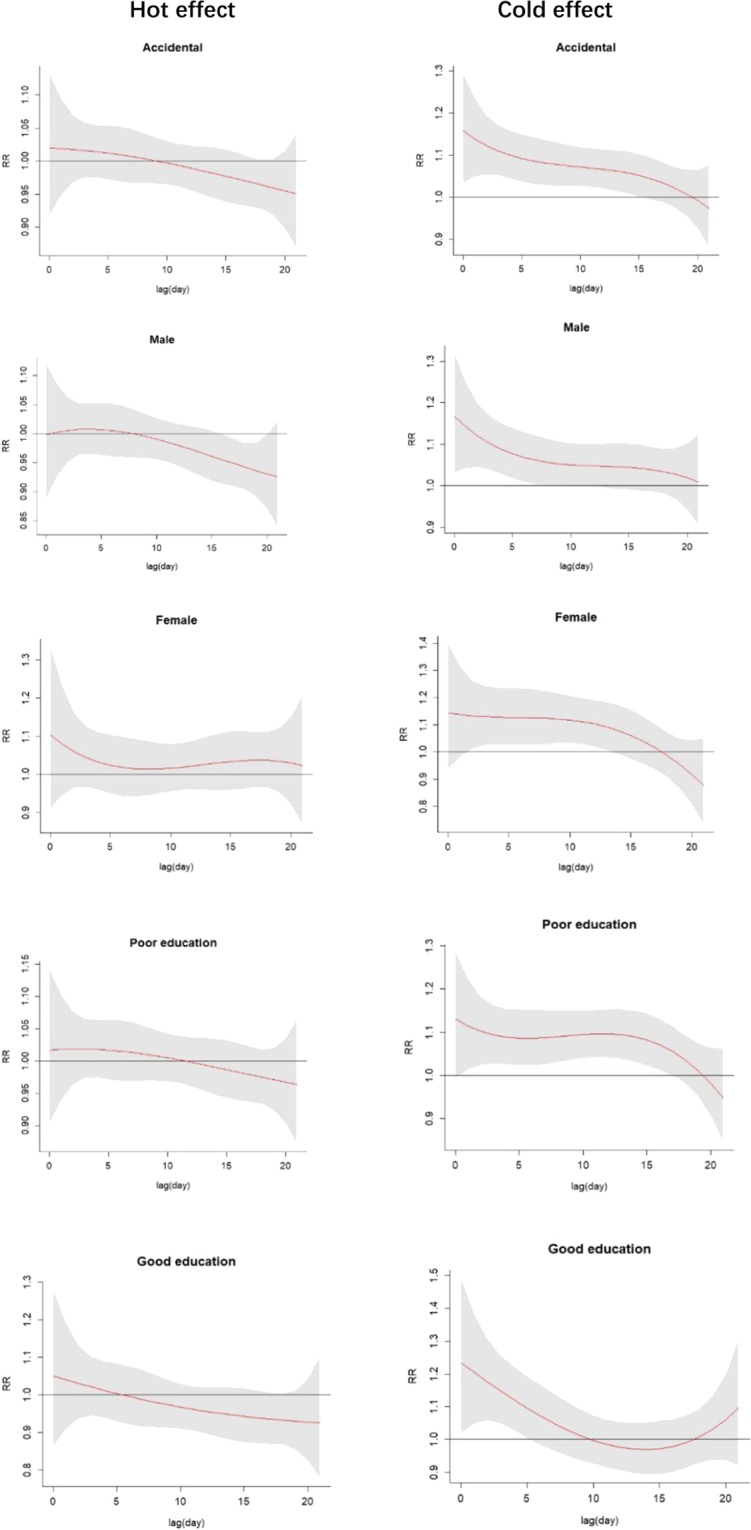
Lag patterns for hot effect (right) and cold effect (left) on accidental human mortality. The red lines are the effect estimates and the grey areas represent the 95% confidential intervals.

The lag patterns for hot and cold effects on accidental human mortality are shown in Fig. 4. Interestingly, a strong and statistically significant cold effects occurred, while hot effects were not correlated significantly with accidental human mortality. The mortality risks of cold effects followed a pattern of increasing RR on the current day or on lag day 2, followed by a decline until lag days 5–17, with lesser effects on subsequent days. This relationship continued to be significant among the observed subgroups stratified by gender and level of education. As shown in Fig. 4, cold effects on all accidents occurred in the range 0–15 d, in the range 0–11 d for males, 2–13 d for females, and 2–17 d for poorly educated people. Interestingly, the lag of the cold effects on well-educated people was relatively short (0–5 d). Specifically, a harvesting effect was observed in the hot effects on males, which is often interpreted as a displacement of deaths that would have occurred in subsequent days regardless of weather changes and may reflect behavioral changes undertaken to reduce exposure to the elements on hot days^17^.

Recent studies have qualitatively confirmed that hot effects are generally short-term in nature, while cold effects last longer^3,18^. Therefore, we calculated the pooled mortality risks of the hot effects at a lag of 0–2 d and cold effects at a lag of 0–21 d stratified by gender and level of education (Table 2). When looking at the pooled hot effects estimate across the varying subgroups, no statistical differences were noted. However, the pooled cold effect estimates were all statistically significant, except for well-educated people, and demonstrated greater RR compared to hot effects among all observed groups. For cold effects, females exhibited the highest pooled effect estimates (RR: 3.14, 95% CI [1.44–6.84]), followed by poorly educated people (RR: 2.63, 95% CI [1.59–4.36]), total accidental human mortality (RR: 2.12, 95% CI [1.36–3.28], males (RR: 1.79, 95% CI [1.10–2.92]), and well-educated people (RR: 1.20, 95% CI [0.58–2.51]). In general, all evidence suggested that cold effects of lag 0-21 days presented a stronger and more significant effect on accidental human mortality compared with hot effects of lag 0-2 days. There was evidence that people were more susceptible to cold effects rather than hot effects, especially females and poorly educated people.

**Table 2:** Pooled mortality risks of hot effect and cold effect.

**Subgroups**	**Hot effect (lag 0-2d)**	**Cold effect (lag 0-21d)**
**Accidental**	0.99(0.71-1.38)	**2.12(1.36-3.28)**
**Gender**		
Male	0.91(0.64-1.31)	**1.79 (1.10-2.92)**
Female	1.29(0.71-2.37)	**3.14 (1.44-6.84)**
**Education attainment**		
Poor education	1.07(0.74-1.55)	**2.63 (1.59-4.36)**
Good education	0.84(0.46-1.55)	**1.20 (0.58-2.51)**

Results are expressed as the relative risk (95% confidence interval).

A sensitivity analysis was carried out to assess the impact of model choices (Table 3). We evaluate changes in the estimated overall effect associated with varying the df used to specify the maximum lag days (14, 21, and 30) and the time trends (7, 8, and 9). Changing the df did not affect the estimates, and the RR was relatively robust. Consequently, we believed that the model choices in our study adequately captured the main effects of temperature on accidental human mortality.

**Table 3:** Sensitivity analysis on the parameters of the model.

**Maximum lag days**	**Cold effects**	**Hot effects**
14	2.15(1.51-3.06)	1.14(0.79-1.64)
21	2.12(1.36-3.28)	0.99(0.71-1.38)
30	2.35(1.37-4.40)	1.05(0.79-1.40)
**df for the time trends**		
7	2.12(1.36-3.28)	0.99(0.71-1.38)
8	2.20(1.38-3.51)	1.05(0.75-1.48)
9	2.29(1.36-3.86)	1.06(0.75-1.50)

Results are expressed as the relative risk (95% confidence interval).

## Discussion

To the best of our knowledge, there is no other study has examined the effects of temperature on accidental human mortality including such a broad range of potential effect confounders in China. We found that the temperature-mortality relationship on accidental human mortality demonstrated an inverted U-shaped curve trend. People were more susceptible to cold effects compared with hot effects, especially females and poorly educated people.

In our analysis, hot effects appeared to have no significant effects on accidental human mortality, a finding is inconsistent with those of the Kim study in Korea^19^. Kim reported that hot temperatures have significant effects on accidental human deaths (RR: 1.03, 95% CI [1.02–1.04]). This difference may have resulted from the different definition of accidental human mortality from our study. We classified accidental human mortality based on ICD 10 code V01-X59; however, Kim classified accidental human mortality based on ICD 10 code V01-Y98, not only including accidents, but also self-harm, assault, and events of undetermined intent.

Heat adaptation may explain why hot effects were not observed in our study. Previous studies have reported that human populations are continually adapting to heat^20^. Several factors, referred to as heat adaptations, may reduce the extent of the impact of hot effects. As noted earlier, Chung reported that economic growth patterns and gross national product growth rate (%) are positively associated with heat adaptation^21^. Shenzhen is one of the economic centers in China and ranked second on the Economist’s 2012 list of the most economically competitive cities in the world. Thus, due to a highly developed economy, people in Shenzhen are likely to adapt to hot temperatures. Second, with the advent and use of air conditioners, the adverse health impacts of heat are decreasing^22,23^. Shenzhen has a high prevalence of air conditioner use, which provides comfortable living and working conditions for people in the summer and tends to prevent hot effects in people. Third, based on a previous global study, people in warmer areas tend to adapt to hot temperatures^1,20^. Shenzhen has a sub-tropical maritime climate, which is mild, with an average annual temperature of 22.4 °C. Therefore, people in Shenzhen have better awareness than many of hot effects on daily life. In addition, government interventions to reduce heat exposure may have also played a role. For example, a law was enacted which requires employers to shorten employee working hours at times of high temperatures, and to pay high-temperature weather subsidies for employees from June to August. All of the above could explain why hot effects were not associated with accidental human mortality in our study.

In contrast, our results clearly demonstrated a stronger relationship between increased risk of accidental human mortality and low temperatures. As discussed previously, low temperature can be a contributing factor in accident and injury causation^24^. The cold effects on accidental human mortality can be understood because people in warm areas poorly adapted to cold weather in the social, physiological and behavioral aspects, while they tend to adapt to hot temperatures^25^. Cold weather can increase the whole-body cooling and decrease the core temperature. Cooling manifests itself with unpleasant sensations and thermal discomfort, which may be a distraction, reducing the performance of tasks requiring concentration and vigilance and may account for the increased incidence of accidental death. Moreover, low temperatures are associated with slower reaction times, which may contribute to the greater risk of accidental human mortality.

Low temperatures are also associated with increased peripheral vasoconstriction, blood pressure, cholesterol, platelet viscosity, and suppressed immune system^1^. These physiological effects could induce the occurrence of chronic diseases^26^ such as cardiovascular diseases and chronic obstructive pulmonary disease, which has been may contribute to an increased risk of accidental death outcomes such as falls, possibly through muscle weakness, functional performance deficit^27^. Additionally, the reduced amount of daylight in the cold days may also play a role through significant effects on human endocrine responses affecting various physiological and psychological responses. These effects can reduce visual perception, and endanger postural stability, which may result in decreased performance and injuries resulting from slipping, tripping or falling accidents^28^. As mentioned above, these physiological effects of the cold may explain large parts of the increased risk of accidental human mortality at low temperatures.

Another contributory factor could be inadequate indoor heating. Since there has been no central heating system in Shenzhen, therefore houses almost entirely lack heating in winter and indoor temperatures are similar to temperatures in the outdoors. Previous studies have found that elderly people are more susceptible to accidents such as falling indoors in winter and such accidents are associated more with cold weather^29–31^. Thus, accidental human mortality may increase at low temperatures as a consequence of the lack of indoor heating. Furthermore, People living in areas with a warmer climate were less likely to prepared for cold days than people living in areas with colder climate, for example, they are not accustomed to wearing hats and gloves, leaving them more susceptible to heat lost^32,33^, as cooling can also be restricted to the extremities (head, hands, and feet). The heat lost may result in lowered mental and physical performance, which may increase the risk of accidents.

Moreover, a cold environment, accompanied by chill, fog, limited visibility, and precipitation, affects people who do not have enough cold-protective measures and could increase the risk of accidents from traffic, falls, slips, and trips. Movement can be hampered, and coordination can be lost by heavy cold-weather clothing, which, in turn, might increase slipping and falling hazards^34^. In summary, low temperature is a risk factor of accidental human mortality. Preventive measures should be considered to reduce cold exposure and minimize the adverse effects of low temperatures.

Long lag times between cold (but not hot) temperature exposure and accidental human mortality were noted in our study, where deaths occurred 11–15 d later. Previous studies have also reported that the cold effects are sustained and lasted for several weeks^35,36^. The shorter times of cold effects in well-educated people persisted for up to 5 d; such individuals are considered to have a higher protection capability and be of an advantaged socio-economic status, and they may be linked to better living conditions and health care services. In addition, such people may have less exposure to low temperatures in their work.

Another conclusion that can be drawn from our results is that females and poorly educated people are more susceptible to cold effects. Previous studies have acknowledged that poorly educated people are more vulnerable to temperature-related mortality^37–39^. Poorly educated People may be subjected to increased exposure to low temperatures and may suffer aggravated vulnerability to low temperatures, which may result from poor living conditions, and a lack of preventive knowledge about precautionary measures for cold exposure. The greater susceptibility of females to low temperatures may be caused by different physiological functions and weaker adaptive capacities. Unfortunately, we did not find a study that has assessed whether the cold effects on accidental human mortality differs between different gender. But an epidemiological analysis, showing a decreased physical performance in older women at low temperatures indoors, provided mixed evidence^40^. Overall, these findings would have implications for cold effect prevention, as well as for public health intervention.

Several caveats to this analysis should be mentioned. We used ambient air temperature data rather than body temperature data. Furthermore, we did not adjust for air pollutants. Finally, due to data constraints and statistical non-significance, we did not take account of other individual level characteristics, such as age, and we avoided reporting case-specific accidental human mortality. Finally, since the analysis was conducted in a single city, the results may vary under different climates. Nevertheless, our study findings may have implications for the future work of temperature-accidental human mortality profile.

## Conclusion

Low temperatures were associated with an increased risk of accidental human mortality, while there were no effects related to hot temperatures. Female and poorly educated people are more susceptible to the effects of low temperatures. Measures preventing cold exposure may have a beneficial effect on accidental human mortality outcomes, particularly in some vulnerable subgroups.

## Data Availability

The data that support the findings of this study are available from Shenzhen Centre for Disease Control and Prevention and Shenzhen Municipal Meteorological Bureau but restrictions apply to the availability of these data, which were used under license for the current study, and so are not publicly available.
